# Recent Sarcopenia Definitions‐Variability in Prevalence and Disability Associations in Peritoneal Dialysis Patients

**DOI:** 10.1002/jcsm.70018

**Published:** 2025-07-29

**Authors:** Sasiwimon Meenetkum, Sarinya Boongird, Piyatida Chuengsaman, Sirinapa Songsrakaew, Sirarat Katesomboon, Kanda Sriudom, Prapimporn Chattranukulchai Shantavasinkul, Wisit Chaveepojnkamjorn, Jiraluck Nontarak, Chagriya Kitiyakara

**Affiliations:** ^1^ Department of Epidemiology, Faculty of Public Health Mahidol University Bangkok Thailand; ^2^ Division of Nephrology, Department of Medicine Faculty of Medicine Ramathibodi Hospital, Mahidol University Bangkok Thailand; ^3^ Banphaeo‐Charoenkrung Peritoneal Dialysis Center, Banphaeo Dialysis Group Banphaeo Hospital Bangkok Thailand; ^4^ Division of Nutrition and Biochemical Medicine, Department of Medicine Faculty of Medicine Ramathibodi Hospital, Mahidol University Bangkok Thailand

**Keywords:** chronic kidney disease, disability, end‐stage kidney disease, muscle function, peritoneal dialysis, sarcopenia

## Abstract

**Background:**

Sarcopenia is common in chronic kidney disease, but no unified consensus exists regarding its diagnostic criteria. New definitions, including the Sarcopenia Definitions and Outcomes Consortium (SDOC), define sarcopenia based on decreased muscle function without measuring muscle mass. However, the application and relationship of newer definitions to functional disability in end‐stage kidney disease, particularly among peritoneal dialysis (PD) patients, remain underexplored. This study evaluated the prevalence and concordance of sarcopenia using older and recent definitions and their association with functional limitations in PD patients.

**Methods:**

This cross‐sectional study evaluated Thai chronic PD patients (*n* = 384) with complete measurements for sarcopenia (BIA, handgrip strength and gait speed). Patients were classified according to the Foundation for the National Institutes of Health (FNIH) Sarcopenia Project, the International Working Group on Sarcopenia (IWGS), the European Working Group on Sarcopenia in Older People 2019 (EWGSOP2), the Asian Working Group for Sarcopenia 2019 (AWGS2019) and the 2020 SDOC. Functional disability was assessed using the Barthel Activities of Daily Living (ADL) score. Associations with dependency were evaluated using multivariable logistic regression.

**Results:**

The median age was 60 years (IQR, 52–68); 54.8% were men, and 31.3% were over 65. Sarcopenia prevalence varied 5‐fold: FNIH (8.3%), IWGS (18.5%), EWGSOP2 (19.0%), AWGS2019 (22.3%) and SDOC (44.5%). Using AWGS2019 as the reference, agreement was good with EWGSOP2 and IWGS but poor with FNIH and SDOC. Of 207 with sarcopenia by any definition, only 15 patients (7.3%) fulfilled all criteria. Sarcopenia prevalence was higher among older adults (≥ 65 years) across all definitions (*p* < 0.001). Functional limitations in at least one domain (ADL ≤ 19) occurred in 86 patients (22.4%) and were more frequent in sarcopenic patients for all definitions except IWGS. By multivariable analysis, only FNIH and SDOC were significantly associated with dependency (ADL ≤ 11): FNIH, OR 5.49, *p* = 0.013; SDOC, OR 6.01, *p* = 0.023. Using AWGS2019 component thresholds, 64.6% had low physical performance, 58.6% had low muscle strength and 22.7% had low muscle mass. Low muscle strength had a higher prevalence of functional limitation (27.1% vs. 15.7%, *p* < 0.05) and dependency (5.80% vs. 0%, *p* = 0.002) than those without.

**Conclusions:**

Sarcopenia is common in PD patients, with substantial variability in prevalence and associations with functional limitations across definitions. Functional impairment was more frequent in low muscle strength. While SDOC was associated with functional deficits, it may overdiagnose sarcopenia in PD. Future studies using clinically relevant outcomes are needed to define sarcopenia in this high‐risk group.

## 1 Introduction

Sarcopenia, the loss of muscle mass and strength due to aging or chronic disease, is associated with increased mortality and poor health outcomes, including functional dependency [1]. Chronic kidney disease (CKD) is a major global health burden, with decreased survival and reduced functional capability [2]. As kidney function declines, multifactorial processes accelerate muscle wasting, resulting in a high prevalence of sarcopenia among patients with end‐stage kidney disease (ESKD) [3]. Peritoneal dialysis (PD) and haemodialysis (HD) are primary modalities of kidney replacement therapy for ESKD patients [2]. Most sarcopenia studies in ESKD have focused on HD, with fewer in PD patients, who may be at special risk due to protein loss via dialysate [4].

Despite being established as a diagnostic entity, there is no consensus on the optimal definition of sarcopenia [5]. Different working groups have proposed varying muscle mass, strength and physical performance combinations with differing cutoffs. Earlier definitions include the Foundation for the National Institutes of Health (FNIH) Sarcopenia Project [6] and the International Working Group on Sarcopenia (IWGS) [7]. New sarcopenia definitions have recently been proposed. In 2019, the European Working Group on Sarcopenia in Older People (EWGSOP2) defined sarcopenia as low muscle mass and low muscle strength [8]. The Asian Working Group for Sarcopenia (AWGS2019) defined this as low muscle mass plus either low muscle strength or low physical performance [9]. Severe sarcopenia encompasses patients with low strength, low lean mass and low physical performance [8]. Unlike other definitions, the 2020 Sarcopenia Definitions and Outcomes Consortium (SDOC) proposed defining sarcopenia solely by low muscle strength and low physical performance without assessing muscle mass, based on evidence that muscle function better predicts falls, mortality and mobility limitations in the elderly [10, 11].

Applying different diagnostic criteria leads to variability in sarcopenia prevalence in the elderly [7]. In CKD, the optimal definition remains unclear, as most studies have used older criteria, and data applying the SDOC definition are limited. Functional limitation, typically measured through basic activities of daily living (ADLs), is a clinically relevant endpoint for evaluating the impact of sarcopenia [12]. In the elderly, muscle strength and performance decline are key drivers of disability progression [6], and functional dependency has been linked to increased mortality in ESKD [8]. However, the relationship between sarcopenia and functional disability, particularly among PD patients, remains underexplored.

Emerging evidence from haemodialysis populations suggests that muscle functional impairment and mitochondrial dysfunction may precede clinical muscle mass loss [13]. Recent reviews highlight that sarcopenia in PD patients involves both muscle mass loss and early declines in strength and performance, reflecting complex pathophysiology distinct from primary aging sarcopenia. Given diagnostic variability across studies and the clinical relevance of functional impairments, evaluating different definitions remains essential in the PD population [14].

This study aims to compare the prevalence and concordance of sarcopenia using older and recent definitions, including SDOC, in PD patients. Additionally, we examine the association between sarcopenia, its components and dependency in activities of daily living [12]. Understanding how different sarcopenia definitions and components relate to functional disability may help optimize identification and management strategies in PD patients.

## 2 Methods

### 2.1 Study Design and Participants

This cross‐sectional study was conducted at Banphaeo General Hospital (Charoenkrung branch), Bangkok, Thailand. All PD patients attending clinic visits between November 1, 2022, and December 31, 2023, were consecutively screened. Eligible participants were aged ≥18 years, had received stable PD for at least 3 months, and could complete bioelectrical impedance analysis (BIA) and blood testing. Exclusion criteria included hospitalization within the past 4 weeks, a planned change in dialysis modality within 6 months, prior kidney transplantation, active cancer treatment, cirrhosis or limb amputation.

The study was approved by the Institutional Review Boards of Ramathibodi Hospital, Mahidol University (COA. MURA #2021/218 and #2023/821) and Banphaeo General Hospital (BGH #012/64) and conducted following the Declaration of Helsinki. Written informed consent was obtained from all participants.

### 2.2 Patient Evaluation and Data Collection

Patient data were collected through interviews and chart reviews. Clinical evaluation and sarcopenia assessment were performed under a standardized protocol after emptying the bladder and draining the peritoneal dialysate. Blood pressure was measured seated after 30 min of rest. Fasting blood samples were collected for complete blood count and biochemical analyses.

Hypertension was defined as SBP ≥ 140 mmHg, DBP ≥ 90 mmHg or use of antihypertensive medications [15]. Diabetes was defined as a history of diabetes, fasting glucose ≥126 mg/dL or medication use [16]. Cardiovascular disease was defined as a history of myocardial infarction, angina, stroke, heart failure, cardiovascular interventions or peripheral vascular disease.

### 2.3 Muscle Mass

Appendicular skeletal muscle mass (ASM) was quantified by BIA using an 8‐electrode multifrequency device (InBody 770, InBody Co. Ltd., Seoul, South Korea). Low muscle mass was defined based on the ASM: BMI ratio for the FNIH and the ASM/height^2^ (Appendicular Skeletal Muscle Mass Index) for other criteria (Table 1).

**TABLE 1:** Sarcopenia and severe sarcopenia diagnostic cutoff values for muscle mass, strength and physical performance by sex according to criteria.

	Low muscle mass (ASM/height^2^)		Low muscle strength		Low physical performance	Diagnosis	
	Men (kg/m^2^)	Women (kg/m^2^)	Men (kg)	Women (kg)	Gait speed (m/s)	Sarcopenia	Severe sarcopenia
AWGS2019	< 7.0	< 5.7	< 28	< 18	< 1.0	LMM + either LMS or LPP	LMM + LMS + LPP
EWGSOP2	< 7.0	< 5.5	< 27	< 16	≤ 0.8	LMM + LMS	LMM + LMS + LPP
IWGS	< 7.23	< 5.67	—	—	< 1.0	LMM + LMS	—
FNIH	ASM/BMI < 0.789	ASM/BMI < 0.512	< 26	< 16	≤ 0.8	LMM + LMS	LMM + LMS + LPP
SDOC	—	—	< 35.5	< 20	< 0.8	LMS + LPP	—

Abbreviations: ASM, appendicular skeletal mass; ASMI, appendicular skeletal muscle index; AWGS2019, the Asian Working Group for Sarcopenia 2019; BMI, body mass index; EWGSOP2, the European Working Group on Sarcopenia in Older People 2; FNIH, the Foundation for the National Institutes of Health; IWGS, the International Working Group for Sarcopenia; LMM, low muscle mass; LMS, low muscle strength; LPP; low physical performance; SDOC, the Sarcopenia Definitions and Outcomes Consortium.

### 2.4 Muscle Strength

Muscle strength was assessed by handgrip strength using the Digital Grip Strength Dynamometer (T.K.K 5401, Takei Scientific Instruments Co., Ltd., Tokyo, Japan). Participants were instructed to hold the dynamometer with one hand, with the arm fully extended and positioned parallel to the body, following the manufacturer's guidelines [12]. Three attempts were performed with the dominant arm after a brief rest period, and the maximum handgrip strength was recorded. If a person could not grip the dynamometer, this was recorded as abnormal handgrip strength.

### 2.5 Physical Performance

Patients were asked to perform the walking test at their usual gait speed with usual walking support. Time to complete 4 m (other criteria) and 6 m (AWGS2019) was recorded using an electronic stopwatch and was used to calculate average gait speed [8]. If a patient could not complete the 6 m within 2 min, this was recorded as low physical performance.

### 2.6 Definitions of Sarcopenia

Sarcopenia was defined by different criteria: AWGS2019, EWGSOP2, FNIH, SDOC, IWGS and severe sarcopenia by AWGS2019, EWGSOP2 and FNIH (Table 1). AWGS2019, used as the standard comparator, defines sarcopenia as follows: (i) low muscle mass (ASM/height^2^: men < 7.0, women < 5.7 kg/m^2^) plus either (ii) low muscle strength (handgrip: men < 28, women < 18 kg) or (iii) low physical performance, for which we used slow gait speed (< 1.0 m/s) or inability to complete the test in 2 min as a surrogate in this study. Severe sarcopenia for AWGS2019, also used as the comparator, requires low ASM, low muscle strength and low physical performance [17].

### 2.7 Functional Limitation and Dependency

Activities of daily living (ADL) were assessed by patient or caregiver interview using the Barthel Activities of Daily Living score, covering 10 domains (each scored 0–2 or 0–3): feeding, bathing, grooming, dressing, toilet use, transfers, mobility, stairs, bowel control and bladder control [18]. Functional limitation was defined as failure to achieve a full score in any domain. The total ADL score (maximum 20) was used to classify functional status, with functional limitation defined as ADL ≤ 19.

Patients were further grouped into four dependency levels: 0–4 (completely dependent), 5–8 (moderately dependent), 9–11 (partially dependent) and 12–20 (independent). Dependency was defined as an ADL score ≤11.

### 2.8 Statistical Analysis

Continuous variables were expressed as mean ± SD or median (25th–75th percentile), depending on normality assessed by the Kolmogorov–Smirnov test, and compared using independent *t* tests or Mann–Whitney *U* tests. Categorical variables were presented as counts (n) and percentages (%) and compared using the Chi‐square test. The prevalence of sarcopenia by each definition was calculated overall and stratified by age group (> 65 and ≤ 65 years) and sex. Agreement between AWGS2019 and other sarcopenia definitions was assessed using Cohen's kappa, with AWGS2019 selected as the reference due to its validation in Asian populations [17]. Risk factors for sarcopenia components (low muscle mass, low muscle strength, and low physical performance) based on AWGS2019 criteria were identified by univariate analysis; variables with *p* < 0.1 or clinical relevance were included in multivariable regression models (enter method). The prevalence of functional limitation and dependency was compared between patients with and without sarcopenia by different definitions and by sarcopenia components using AWGS2019 cutoffs. Associations between sarcopenia and ADL dependency were analysed by logistic regression. Odds ratios (ORs) and 95% confidence intervals (CIs) were calculated after adjustment for age, sex, diabetes and dialysis vintage, based on prior associations with muscle health in dialysis populations [19, 20, 21]. All analyses were performed using SPSS version 11.5 (SPSS Inc., Chicago, Illinois). A two‐sided *p* value < 0.05 was considered statistically significant.

## 3 Results

### 3.1 Patient Characteristics

Of the 414 PD patients invited to participate, 30 were excluded for the following reasons: declined (*n* = 15), withdrew (*n* = 2), inability to undergo the BIA test (*n* = 10) or draw blood (*n* = 1), or life expectancy less than 6 months (*n* = 2) (Figure S1). Of 384 included patients, 54.8% were men and 31.3% were over 65 years old. Patient characteristics are shown in Table 2. The median age was 60 years. Over half were married (51.9%), and 5.2% lived alone. Over half (54.2%) had primary school or lower education. Hypertension (89.8%) and diabetes mellitus (70.6%) were common. The median PD vintage was 17.6 months.

**TABLE 2:** Patient characteristics.

Characteristics	All cases	MEN	WOMEN	*p* value
	(*n* = 384)	(*n* = 211)	(*n* = 173)	
**Demographic data**				
Age (years)	60.0 (52.0, 67.8)	59.0 (51.0, 68.0)	61.0 (52.0, 67.0)	0.285
Weight (kg)	59.6 (52.2, 68.2)	62.7 (56.1, 71.3)	56.1 (48.5, 64.0)	**< 0.001** *
BMI (kg/m^2^)	23.0 (20.8, 26.0)	22.9 (20.8, 25.6)	23.1 (11.5, 45.5)	0.620
Married, *n* (%)	227 (51.9)	131 (62.1)	96 (55.5)	0.211
Live alone, *n* (%)	20 (5.2)	14 (6.6)	6 (3.5)	0.258
Education level, *n* (%)				
Primary school or lower	208 (54.2)	96 (45.5)	112 (64.7)	**0.001** *
Secondary or vocational	125 (32.6)	80 (37.9)	45 (26.0)	
Bachelor's degree or above	51 (13.3)	35 (16.6)	16 (2.9)	
Comorbidity, *n* (%)				
Hypertension	345 (89.8)	191 (90.5)	154 (89.0)	0.627
Diabetes mellitus	272 (70.6)	158 (74.9)	113 (65.3)	**0.043** *
Cardiovascular disease	180 (46.8)	109 (51.7)	71 (41.0)	**0.040** *
Peritoneal dialysis vintage (month)	17.6 (8.5, 41.9)	15.2 (6.7, 33.7)	23.1 (11.5, 45.5)	**0.002** *
**Laboratory parameters**				
Haemoglobin (*n* = 378)	10.4 (9.0, 11.6)	10.4 (9.3, 11.8)	10.2 (8.5, 11.4)	0.054
Serum C‐reactive protein (mg/L)	1.5 (0.5, 4.7)	1.5 (0.5, 4.7)	1.4 (0.4, 4.8)	0.638
Serum bicarbonate (mmol/L)	24.3 (22.0, 26.6)	23.8 (21.8, 26.3)	24.8 (22.2, 26.8)	0.080
Serum blood urea nitrogen (mg/dL)	44.3 (26.3, 60.0)	47.7 (23.6, 62.8)	41.1 (30.9, 54.4)	0.286
Serum creatinine (mg/dL)	8.8 (4.1, 11.4)	9.0 (4.2, 12.9)	8.5 (4.1, 10.2)	**0.049** *
Serum albumin (mg/dL)	33.9 ± 7.7	33.5 ± 8.8	34.0 ± 7.4	0.679
Serum Parathyroid hormone (pg/mL)	166 (77, 317)	173 (87, 318)	156 (71, 305)	0.509
Cholesterol (mg/dL)	193 (158, 244)	185 (150, 235)	204 (169, 257)	**0.013** *
Total Kt/V_urea_ (*n* = 363)	2.1 ± 0.7	2.2 ± 1.0	2.5 ± 0.9	**< 0.001** *
Peritoneal_Kt/V_urea_ (*n* = 360)	1.7 (1.5, 2.0)	1.6 (1.4, 1.8)	2.0 (1.8, 2.2)	**< 0.001** *
nPNA (g/kg/day) (*n* = 355)	1.1 (0.9, 1.3)	1.0 (0.9, 1.2)	1.1 (1.0, 1.3)	**0.002** *
**Sarcopenia components**				
ASM (kg)	19.1 (15.7, 22.8)	21.7 (18.9, 24.6)	16.3 (13.6, 18.6)	**< 0.001** *
ASMI (kg/m^2^)	7.4 (6.5, 8.3)	7.8 (7.0, 8.8)	6.8 (5.9, 7.6)	**< 0.001** *
ASM:BMI ratio	0.8 (0.7, 1.0)	1.0 (0.9, 1.1)	0.7 (0.6, 0.8)	**< 0.001** *
Handgrip strength (kg) (*n* = 379^a^)	21.4 (16.5, 27.6)	26.2 (21.5, 31.1)	17.2 (14.2, 20.4)	**< 0.001** *
Gait speed (m/s) (*n* = 301^a^)	0.9 (0.7, 1.5)	0.9 (0.7, 1.5)	0.9 (0.7, 1.6)	0.383
**Low muscle mass by ASM/height** ^**2**^ (kg/m^2^), *n* (%)				
AWGS2019 (men < 7.0, women < 5.7)	87 (22.7)	49 (23.2)	38 (22.0)	**< 0.001** *
EWGSOP2 (men < 7.0, women < 5.5)	79 (20.6)	49 (23.2)	30 (17.3)	**< 0.001** *
IWGS (men < 7.23, women < 5.67)	93 (24.2)	58 (27.5)	35 (20.2)	**< 0.001** *
**Low muscle mass by ASM: BMI**, *n* (%)				
FNIH (men < 0.789, women < 0.512)	54 (14.1)	40 (19.9)	14 (8.1)	**0.006** *
**Low muscle strength**, *n* (%)^b^				
AWGS2019 (men < 28, women < 18)	225 (58.6)	126 (59.7)	99 (57.2)	**< 0.001** *
EWGSOP2 (men < 27, women < 16)	189 (49.2)	118 (55.9)	71 (41.0)	**< 0.001** *
FNIH (men < 26, women < 16)	173 (45.0)	102 (48.3)	71 (41.0)	**< 0.001** *
SDOC (men < 35.5, women < 20)	331 (86.2)	186 (88.2)	125 (72.3)	**< 0.001** *
**Low physical performance**, *n* (%)^c^				
Gait speed < 0.8 m/s	198 (51.6)	105 (49.8)	93 (53.8)	0.473
Gait speed ≤ 0.8 m/s	199 (51.8)	105 (49.8)	94 (54.3)	0.412
Gait speed < 1.0 m/s	249 (64.8)	133 (63.0)	116 (67.1)	0.453

*Note:* Data are shown as mean ± SD; median (25,75 percentile); *n* (%). Total Kt/VUrea = the sum of weekly peritoneal and urine urea clearances; Peritoneal Kt/V_Urea_ represented weekly peritoneal urea clearances.

Abbreviations: ASM, appendicular skeletal mass; ASMI, appendicular skeletal muscle index; AWGS2019, the Asian Working Group for Sarcopenia 2019; BMI, body mass index = weight (kg)/height^2^ (m); EWGSOP2, the European Working Group on Sarcopenia in Older People 2; FNIH, the Foundation for the National Institutes of Health; IWGS, the International Working Group for Sarcopenia; nPNA, normalized protein nitrogen appearance; SDOC, the Sarcopenia Definitions and Outcomes Consortium.

* *p* value < 0.05 men versus women.

^a^ *n* = those able to perform the test (all 384 patients attempted the test).

^b^ Low muscle strength = unable to perform or low handgrip strength (kg) by each criterion;

^c^ Low physical performance = unable to complete walk in allotted time or slow gait speed (m/s) by each criteria.

Men and women had similar ages. Five patients could not perform the handgrip test and were classified as having low muscle strength across all definitions. Despite higher absolute muscle mass and handgrip strength values, men were more likely to meet the low muscle mass and strength criteria than women. Eighty‐three (21.6%) patients could not complete the gait speed test (non‐completers) within the allotted time and were classified as having low physical performance across all sarcopenia definitions. Rates of low physical performance did not differ between men and women at any cutoff point. Table S1 compares patients who completed the gait speed test (*n* = 301) with those who did not. Non‐completers were older, with higher cardiovascular disease rates and lower serum PTH levels. While muscle mass did not differ significantly between groups, non‐completers had a higher prevalence of low muscle strength across nearly all definitions.

Patient characteristics stratified by the presence or absence of sarcopenia by the AWGS2019 definition are presented in Table S2. Sarcopenic patients were older, had lower BMI, income, serum creatinine and cholesterol, and had a higher prevalence of cardiovascular disease.

### 3.2 Prevalence of Sarcopenia

**FIGURE 1:**
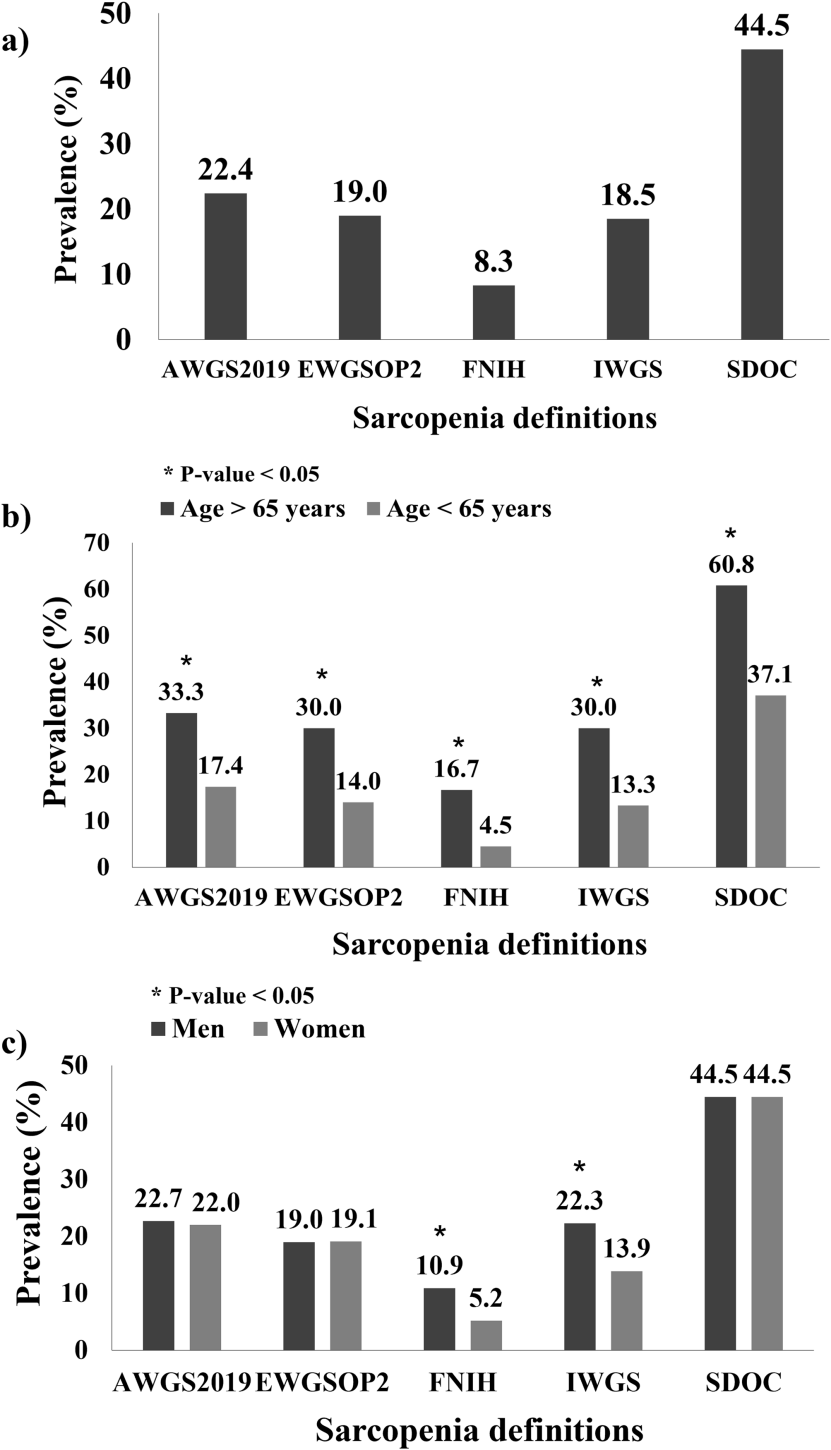
Prevalence of sarcopenia by different definitions. (a) All patients (*n* = 384); (b) by age: ≥ 65 years (*n* = 120), < 65 years (*n* = 264); and (c) by sex: men (*n* = 211), women (*n* = 173). *p value < 0.05.

Sarcopenia prevalence varied nearly 5‐fold across definitions, from 8.3% (FNIH) to 44.5% (SDOC), with intermediate rates for IWGS (18.5%), EWGSOP2 (19.0%) and AWGS2019 (22.4%) (Figure 1a). As shown in Table 3, agreement with AWGS2019 was good for IWGS and EWGSOP2, but poor for FNIH and SDOC. Positive Percent Agreement (PPA) ranged from 32.2 to 91.5%, while Negative Percent Agreement (NPA) remained consistently high across all definitions. Among patients completing the gait speed test (*n* = 301), sarcopenia prevalence varied across definitions in a similar trend, but consistently remained lower than in the overall cohort: FNIH (8.3%), IWGS (15.3%), EWGSOP2 (17.9%), AWGS2019 (21.6%) and SDOC (33.2%).

**TABLE 3:** Agreement of sarcopenia diagnosis between AWGS2019 and other diagnostic criteria (*n* = 384).

Diagnosis criteria	Overall		PPA (%)	NPA (%)	K^a^ (95% CI)	*p* value
	Sarcopenia	None				
**AWGS2019 (ref.)**	86 (22.3%)	298 (77.4%)				
**EWGSOP2**	73 (19.0%)	311 (80.8%)	89.0	93.2	**0.77 (0.69–0.85)**	**< 0.001** *
**FNIH**	32 (8.3%)	352 (91.4%)	68.8	81.8	**0.29 (0.17–0.39)**	**< 0.001** *
**IWGS**	71 (18.4%)	313 (81.3%)	91.5	93.3	**0.78 (0.70–0.86)**	**< 0.001** *
**SDOC**	171 (44.5%)	213 (55.5%)	32.2	85.4	**0.19 (0.09–0.28)**	**< 0.001** *

*Note:* The kappa value represents good (> 0.75); moderate (0.40–0.75); poor (< 0.40) agreement beyond chance.

Abbreviations: AWGS2019, the Asian Working Group for Sarcopenia 2019; EWGSOP2, the European Working Group on Sarcopenia in Older People 2; FNIH, the Foundation for the National Institutes of Health; IWGS, the International Working Group for Sarcopenia; NPA, negative predictive value; PPA, positive predictive value; SDOC, the Sarcopenia Definitions and Outcomes Consortium.

* *p* < 0.05.

### 3.3 Patient Concordance Across Definitions

Sarcopenia was identified by at least one definition in 207 participants (53.9% of all). Figure S2 illustrates the distribution and overlap of sarcopenia diagnoses across the five criteria. Only 15 patients (7.2% of sarcopenic) met all five definitions, reflecting poor overall concordance. The highest overlap occurred between AWGS2019 and EWGSOP2, with 65 patients (31.4%) fulfilling both. Notably, 43.3% were classified as sarcopenic by SDOC alone without meeting other criteria.

### 3.4 Prevalence of Sarcopenia by Age and Sex

In the age group over 65 years, the prevalence ranged from 60.8% (SDOC) to 16.7% (FNIH), with AWGS 2019 showing an intermediate value (33.3%). For those under 65 years, the prevalence ranged from 37.1% (SDOC) to 4.5% (FNIH), with AWGS2019 being 17.4% (Figure 1b). Sarcopenia prevalence was higher in older individuals compared to those under 65 for all criteria (*p* < 0.001). The prevalence ratios (age over 65 to under 65) for AWGS2019, EWGSOP2, FNIH, IWGS and SDOC were 1.9, 2.1, 3.7, 2.3 and 1.6, respectively.

The prevalence of sarcopenia was similar in men and women for AWGS2019, EWGSOP2 and SDOC (Figure 1c). However, the prevalence was significantly higher in men for IWGS (*p* = 0.044) and FNIH (*p* = 0.035). In men, the prevalence ranged from 44.5% (SDOC) to 10.9% (FNIH), and among women, the prevalence ranged from 44.5% (SDOC) to 5.2% (FNIH). The ratio for women to men for AWGS2019, EWGSOP2, FNIH, IWGS and SDOC was 1.0, 0.5, 0.6 and 1.0, respectively.

### 3.5 Sarcopenia and Functional Limitation

#### 3.5.1 Any Functional Limitation

**FIGURE 2:**
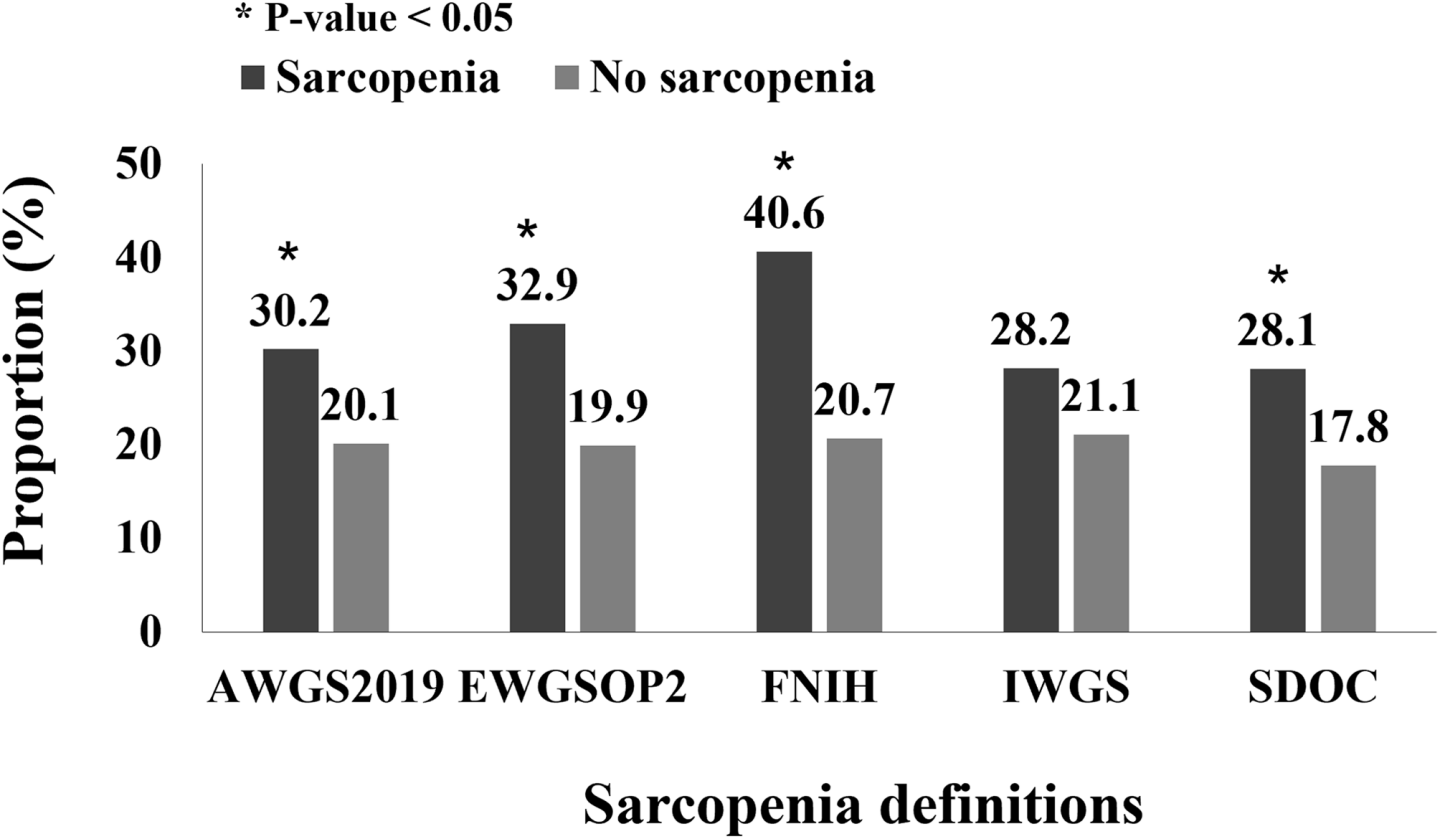
Proportion of patients with any functional limitation, stratified by sarcopenia status according to different definitions (*n* = 384). Functional limitation was defined as a Barthel Activities of Daily Living score ≤ 19.

Eighty‐six patients (22.4%) had functional limitations in at least one domain (Barthel ADL score ≤19). The prevalence of functional limitations was consistently higher among patients with sarcopenia compared to those without, across all definitions except IWGS (Figure 2).

#### 3.5.2 Functional Limitations in Individual ADL Domains

**FIGURE 3:**
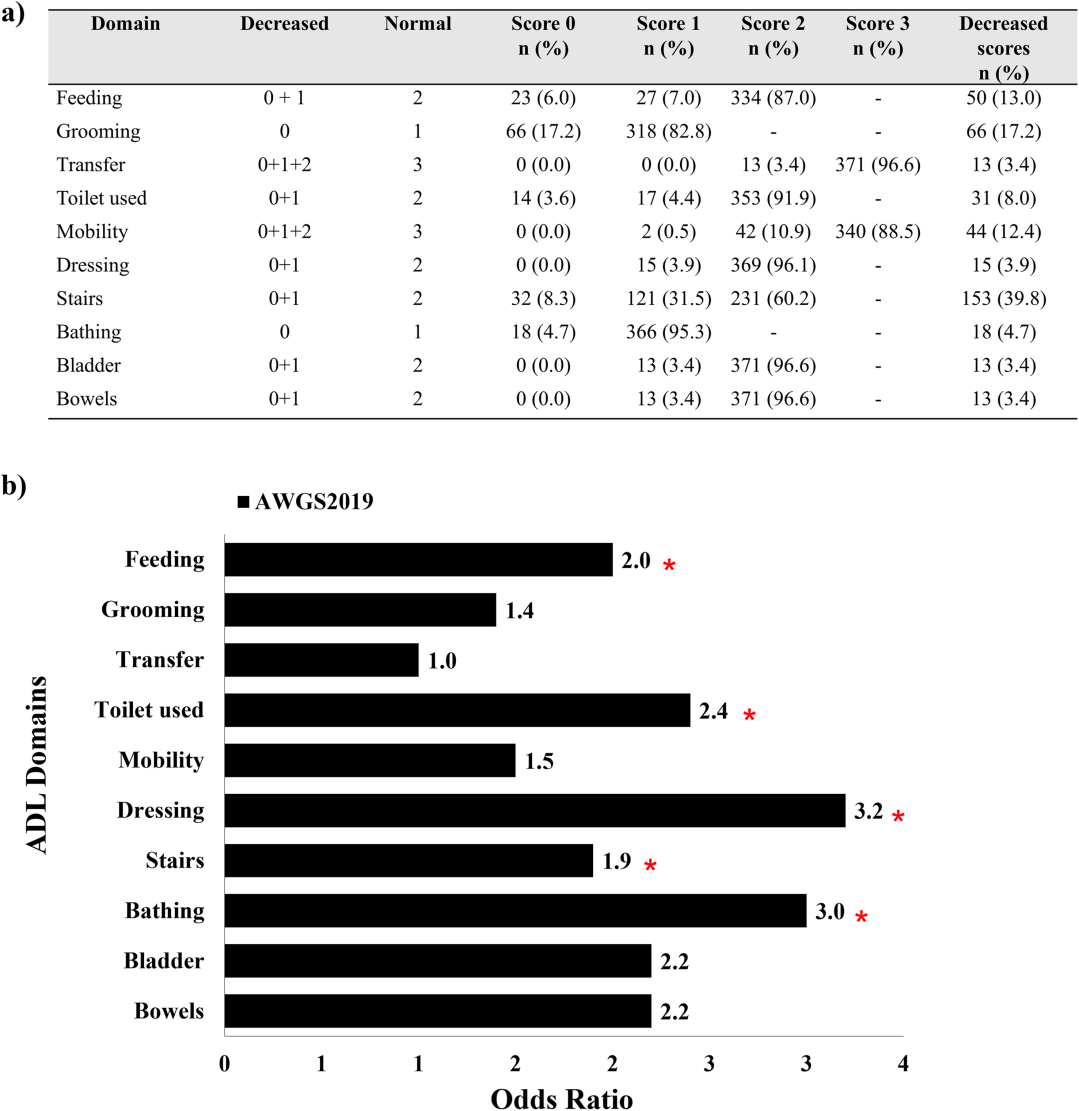
Association between sarcopenia (AWGS2019) and individual activities of daily living (ADL) in PD patients (*n* = 384). (a) Distribution of Barthel scores and proportion of patients with reduced scores across ADL domains. (b) Odds ratios for functional impairment in each ADL domain associated with sarcopenia, defined by AWGS2019 criteria. Functional impairment was defined as a score below the full mark in each domain. Each bar compares patients with sarcopenia to those without (reference group). *p < 0.05.

Figure 3 shows the distribution of Barthel ADL scores across domains. Stair climbing was the most frequently impaired activity (39.8%), while basic activities such as dressing and bathing showed low impairment rates (< 5%). Compared to non‐sarcopenic patients, those with sarcopenia (AWGS2019 definition) had significantly higher odds of functional limitation in dressing (OR 3.2, *p* = 0.029), bathing (OR 2.9, *p* = 0.027), toilet use (OR 2.4, *p* = 0.027), feeding (OR 2.0, *p* = 0.037) and stair climbing (OR 1.9, *p* = 0.008). Variability in the pattern and magnitude of ADL limitations was observed across different sarcopenia definitions (Figure S3). FNIH and SDOC criteria demonstrated broader associations with ADL impairment and higher mean odds ratios compared to AWGS2019, EWGSOP2 and IWGS.

#### 3.5.3 Sarcopenia and Dependency

The patients were classified according to the Barthel ADL Index as follows: completely dependent (*n* = 0), moderately dependent (*n* = 7), partially dependent (*n* = 6) and independent (*n* = 371) [18]. By univariate analysis, sarcopenia by FNIH and SDOC was significantly associated with an increased risk of any dependency (ADL ≤ 11, *n* = 13) (Table 4). After adjusting for sex, age, diabetes and dialysis vintage, sarcopenia by FNIH and SDOC, but not others, was independently associated with dependency: (OR: FNIH 5.49 [*p* = 0.013*]; SDOC 6.01 [*p* = 0.023]).

**TABLE 4:** Association of dependency (ADL ≤ 11) according to sarcopenia criteria.

Sarcopenia criteria	ADL dependency status, *n* (%)		Uni OR (95% CI)	*p* value	Multi OR (95% CI)	*p* value
	Independent	Low to moderately dependent				
**AWGS2019** Sarcopenia	82 (95.3)	4 (4.7)	1.57 (0.47–5.22)	0.465	1.33 (0.39–4.57)	0.654
No sarcopenia	289 (97.0)	9 (3.0)				
**EWGSOP2** Sarcopenia	68 (93.2)	5 (6.8)	2.79 (0.88–8.78)	0.080	2.43 (0.74–7.91)	0.142
No sarcopenia	303 (97.4)	8 (2.6)				
**FNIH** Sarcopenia	28 (87.5)	4 (23.5)	**5.44** **(1.58–18.80)**	**0.007** *	**5.49** **(1.43–21.06)**	**0.013** *
No sarcopenia	343 (97.4)	9 (2.6)				
**IWGS** Sarcopenia	68 (95.8)	3 (4.2)	1.34 (0.36–4.99)	0.666	1.28 (0.32–5.07)	0.727
No sarcopenia	303 (96.8)	10 (3.2)				
**SDOC** Sarcopenia	160 (93.6)	11 (6.4)	**7.25** **(1.59–33.18)**	**0.011** *	**6.01** **(1.28–28.24)**	0.023*
No sarcopenia	211 (99.1)	2 (0.9)				

*Note:* Multivariable adjustments for age, sex, diabetes and dialysis vintage.

Abbreviations: ADL, activities of daily living; AWGS2019, the Asian Working Group for Sarcopenia 2019; EWGSOP2, the European Working Group on Sarcopenia in Older People 2; FNIH, the Foundation for the National Institutes of Health; IWGS, the International Working Group for Sarcopenia; SDOC, the Sarcopenia Definitions and Outcomes Consortium.

* *p* < 0.05.

#### 3.5.4 Severe Sarcopenia

##### 3.5.4.1 Prevalence

The prevalence of severe sarcopenia was 13.8% by AWGS2019, 12.8% by EWGSOP2 and 6.0% by FNIH criteria (Figure S4a). The ratio of severe sarcopenia to overall sarcopenia ranged from 0.6 to 0.7 across definitions. The agreement between AWGS2019 and EWGSOP2 was good, but it was poor with FNIH (Table S3). Severe sarcopenia was significantly more common in older than younger patients (*p* < 0.001; Figure S4b), with no significant difference between men and women (Figure S4c).

##### 3.5.4.2 Association With Functional Limitation and Dependency

When sarcopenia was categorized into three groups (no sarcopenia, sarcopenia and severe sarcopenia), a graded increase in the proportion with functional limitation was observed with EWGSOP2 criteria but not with AWGS2019 or FNIH (Figure S5). Due to the small number of patients with dependency, differences between the three groups for dependency were not evaluated.

##### 3.5.4.3 Sarcopenia Components

Using AWGS2019 criteria, 22.7% of patients had low muscle mass, 58.6% had low muscle strength and 64.8% had low physical performance.

##### 3.5.4.4 Factors Associated With Sarcopenia Components

In multivariate analysis (Table S4), low muscle mass was associated with older age (OR 1.04, *p* = 0.003), lower BMI (OR 0.64, *p* < 0.001) and absence of hypertension (OR 0.24, *p* = 0.003) (Table S4). Low physical performance was linked only to increasing age (OR 1.03, *p* < 0.001). Low muscle strength was associated with older age (OR 1.05, *p* < 0.001), lower BMI (OR 0.94, *p* = 0.016), diabetes (OR 1.70, *p* = 0.038) and cardiovascular disease (OR 1.70, *p* = 0.020). Sex, dialysis vintage and nutritional parameters showed no significant associations with any sarcopenia component. The association between low muscle strength and serum albumin was not significant after adjustment.

##### 3.5.4.5 Sarcopenia Components and Functional Limitation

**FIGURE 4:**
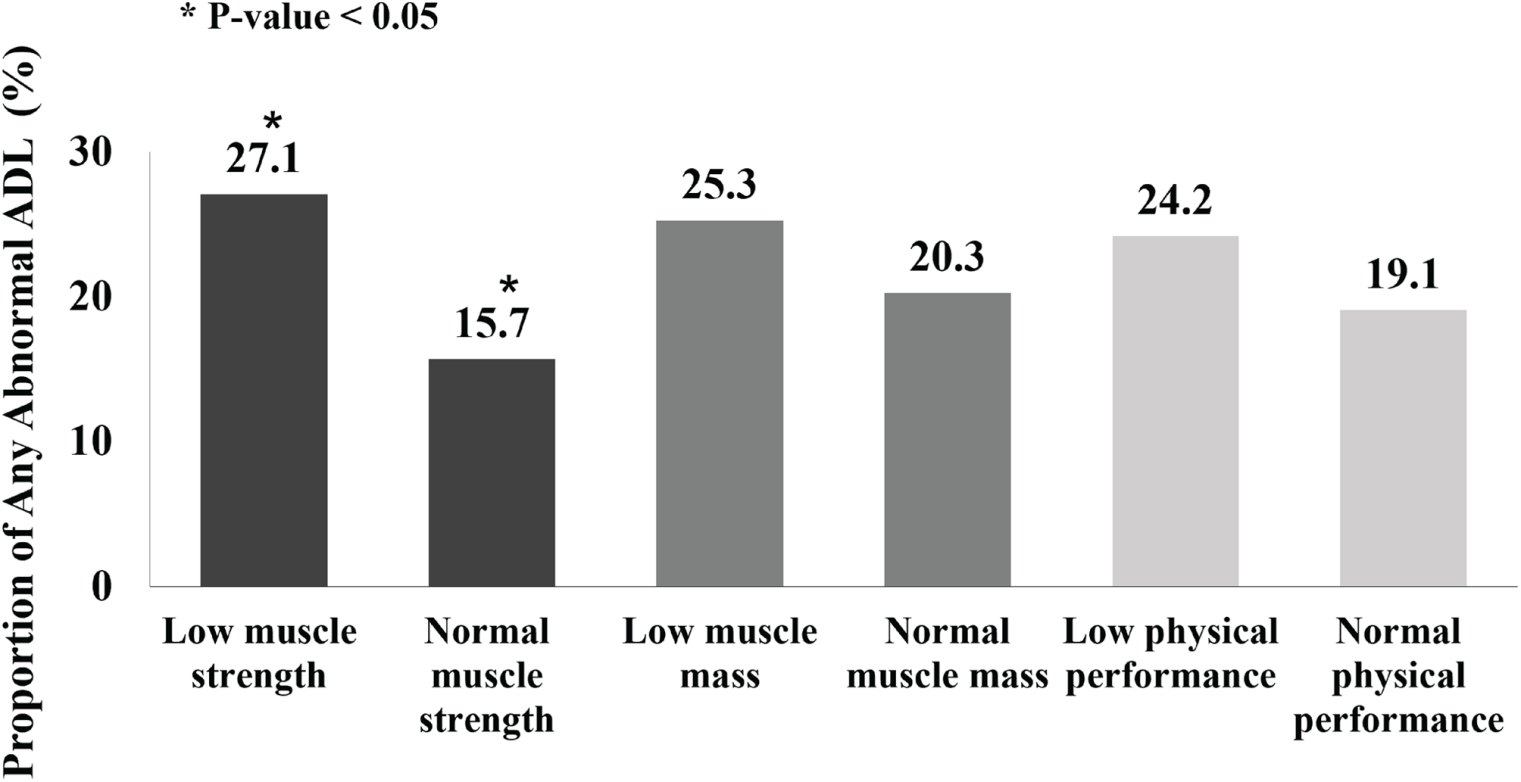
Proportion of patients with any functional limitation, stratified by sarcopenia components according to AWGS2019 cutoff criteria (*n* = 384). Functional limitation was defined as a Barthel Activities of Daily Living (ADL) score ≤ 19.

Figure 4 shows that functional limitation (ADL score ≤19) was more common in patients with low muscle strength (27.1%) compared to those with normal strength (15.7%, *p* < 0.05), while no significant differences were seen for low versus normal muscle mass or physical performance.

Table S5 shows that dependency (ADL score ≤11) was significantly more frequent in patients with low muscle strength (*p* < 0.05). Although dependency rates were numerically higher in those with low muscle mass or low physical performance, the differences were not statistically significant.

## 4 Discussion

This study investigated the prevalence of sarcopenia using contemporary definitions and its association with disability in 384 Thai PD patients. A striking finding was the substantial variability in sarcopenia prevalence, ranging 5‐fold from 8.3% (FNIH) to 44.5% (SDOC), highlighting poor agreement across diagnostic criteria. SDOC identified sarcopenia in nearly two‐thirds of older PD patients and a considerable proportion of younger individuals. Furthermore, AWGS2019 criteria revealed a high prevalence of low physical performance (over two‐thirds) and muscle strength (over half), with low muscle strength emerging as a more consistent predictor of functional limitation than muscle mass or physical performance. While most definitions linked sarcopenia to functional limitation, only SDOC and FNIH independently predicted dependency after multivariable adjustment, each conferring a 5–6 fold increased risk. Future longitudinal studies focusing on clinical outcomes are crucial to establish optimal sarcopenia definitions for PD.

Only 15 patients (7.3% of those with sarcopenia by any definition) met criteria across all definitions. These large intra‐individual discrepancies reflect differences in the cutoff thresholds for muscle mass, handgrip strength or gait speed and the varying combinations required in different criteria [22, 23]. EWGSOP2 and IWGS showed better concordance with AWGS2019, likely due to the shared requirement for low muscle mass, with one abnormal functional test, albeit with variations in cutoff points and combinations. EWGSOP2 required both low muscle mass and low muscle strength; AWGS2019 required the combination of low muscle mass and either low muscle strength or low physical performance. In contrast, IWGS required the combination of low muscle mass and low physical performance for the diagnosis.

FNIH criteria, using BMI‐adjusted muscle mass instead of height and a lower muscle strength threshold, produced the lowest prevalence [6]. The difference in the normalization methods likely accounts for the poor agreement with AWGS2019. Our findings are consistent with a previous study in community‐based Chinese women, which showed similarities between AWGS2019, EWGSOP2 and IWGS and poor agreement with FNIH [24].

Developed to predict adverse outcomes such as falls and mobility loss in the elderly, the SDOC criteria focus solely on low muscle strength and low physical performance [11, 23, 25, 26, 27]. Few prior studies have applied SDOC criteria outside elderly community cohorts. The exclusion of muscle mass, a cornerstone of other definitions, combined with a higher muscle strength threshold, likely accounts for the strikingly higher prevalence of sarcopenia and poor concordance with other criteria observed in our study. Our findings in PD patients contrast with observations from a European cohort of community‐dwelling older adults [28]. In that study, SDOC (2.0%) identified fewer cases than IWGS (3.6%) but more than EWGSOP2 and AWGS2019 (0.7% for both). Low concordance between SDOC and other criteria was also reported, confirming the definitional inconsistency we observed, although with different prevalence patterns [28].

Sarcopenia risk factors may differ between HD and PD. PD involves continuous exposure to glucose‐based dialysate, leading to greater glucose absorption and protein loss, whereas HD uses intermittent dialysis sessions to clear waste [2]. A systematic review and meta‐analysis reported a pooled sarcopenia prevalence of 25.6% (95% CI 22.1–29.4%) [29]. However, research specifically assessing sarcopenia prevalence in PD patients remains limited, often relying on outdated diagnostic criteria [30, 31]. A few recent studies have applied updated definitions such as EWGSOP2 or AWGS2019, but none have evaluated SDOC. Among 50 Brazilian PD patients, sarcopenia prevalence was 10% using EWGSOP2, compared to 4% with the original 2010 EWGSOP definition [32]. In a multi‐ethnic PD cohort from the United Kingdom, sarcopenia prevalence was 17.7% using AWGS2019 or EWGSOP2, comparable to our findings [33]. A study of 186 Taiwanese PD patients reported sarcopenia prevalence rates of 38.2%, 34.9%, 31.2% and 25.8% according to AWGS2019, IWGS, EWGSOP2 and FNIH, respectively [34], with higher rates possibly reflecting longer dialysis vintage in that cohort.

Sarcopenia (usually by older definitions) has been associated with disability in older adults [35]. Severe sarcopenia, but not sarcopenia by AWGS2019 or EWGSOP2 criteria, was associated with a 4‐fold risk of dependency in Chinese HD patients [36]. So far, there is limited information on the association of sarcopenia with functional ability in PD. Our study extends previous observations by showing that the strength of association between sarcopenia and ADL impairment varies depending on the diagnostic definition. FNIH and SDOC demonstrated the strongest links to dependency after adjustment. Our findings are consistent with previous studies in that SDOC has been shown to identify older adults at risk of losing independence [37]. Although the presence of PD catheters could theoretically impair ADL tasks like dressing, these activities were minimally affected in our cohort. Sarcopenic patients most frequently demonstrated limitations in stair climbing, bathing, dressing and toilet use. These patterns of ADL limitations in PD patients may differ from those observed in HD patients, emphasizing the need for further comparative studies.

Several factors in CKD and PD patients contribute to the complex interplay of pathophysiological mechanisms leading to muscle wasting and weakness [3, 14]. In our study, analysis of sarcopenia components further revealed that low muscle strength was more strongly associated with functional impairment than low muscle mass. This aligns with evidence suggesting muscle strength and function decline earlier than mass in haemodialysis patients [13]. Chronic inflammation, uremic toxins, oxidative stress, mitochondrial dysfunction, and protein‐energy wasting inherent to CKD and dialysis may impair neuromuscular integrity and endurance before measurable reductions in muscle quantity occur [14]. Thus, strength‐focused assessments may be more sensitive markers of early muscle dysfunction and impending disability in the PD population. Our data is consistent with a previous study showing that muscle strength was a better predictor of survival than muscle mass in PD patients [38].

Although sarcopenia cutoff criteria were initially developed for the elderly, our findings show considerable prevalence even among PD patients under 65 years old, ranging from 4.5 to 37.1%. Our findings highlight the compounded impact of CKD pathology and dialysis‐related factors on muscle health. In contrast, a meta‐analysis of community‐based cohorts over 65 reported much lower sarcopenia prevalence, 1.1% by EWGSOP2 and 1.7% by SDOC [23], even when compared to our older PD cohort. Sex‐specific thresholds are included in all sarcopenia criteria. Our study showed no significant sex differences in sarcopenia prevalence when using recent definitions, but older definitions (IWGS, FNIH) showed higher prevalence in men. These discrepancies likely reflect improvements in cutoff points derived from more representative data in newer criteria.

Using AWGS2019 criteria, over two‐thirds of patients had low physical performance, over half had low muscle strength, and 23% had low muscle mass. Older age remained the strongest predictor across all sarcopenia components, consistent with previous studies [19, 20, 21]. The variability of other risk factors across studies likely reflects differences in diagnostic criteria and assessment methods. Consistent with Kang et al., dialysis vintage, adequacy and nutritional markers showed no significant associations with sarcopenia components [17]. Our findings that lower BMI and comorbidities such as diabetes and cardiovascular disease were associated with reduced muscle strength but not consistently with low muscle mass raise the possibility that pathogenic mechanisms may impact muscle mass and function differently in PD patients.

We also explored sarcopenia severity based on EWGSOP2, AWGS2019 and FNIH criteria. Approximately two‐thirds of sarcopenic patients had severe sarcopenia, with moderate concordance between definitions. A graded association between severity and functional limitation was observed only with EWGSOP2, suggesting that the sequential addition of impairments in gait speed after low muscle strength enhances the identification of patients at highest functional risk.

Our study has several important implications. The high prevalence of sarcopenia and its association with dependency emphasizes the urgent need for improved identification, understanding and management of sarcopenia in CKD. Clinicians must recognize that sarcopenia diagnosis varies substantially depending on the definition used, leading to inconsistent identification and influencing treatment strategies. Optimal diagnostic criteria and cutoff values for CKD and ESKD populations remain undefined. Although AWGS2019 was anticipated to suit our Thai PD population, it was not associated with functional dependency in this study. In contrast, FNIH criteria identified a smaller group but showed stronger associations with dependency. This suggests that BMI may better reflect an individual's muscle mass relative to body size, and fat content adjustment, which may better reflect functional impairment [39]. Our findings showed that low muscle strength and performance were far more prevalent than low muscle mass under AWGS2019. Moreover, low muscle strength, but not low muscle mass, was consistently associated with functional limitations. Risk factors for low muscle mass and low muscle strength also differed, with only older age common to both, supporting evidence that pathogenic mechanisms may differentially affect muscle mass and function in CKD, with earlier impacts on strength contributing to disability. Unlike muscle mass‐based definitions, SDOC focuses entirely on muscle strength and physical performance, offering practical advantages by directly capturing functional deficits without requiring specialized equipment. However, SDOC diagnosed a substantially higher proportion of PD patients than other criteria, raising concerns about potential overdiagnosis. Given the distinct pathophysiology of muscle dysfunction in CKD and ESKD, caution is needed when applying SDOC thresholds validated in healthy elderly populations to dialysis cohorts. Longitudinal studies are necessary to validate strength‐ and function‐based sarcopenia definitions and establish clinically meaningful thresholds for ESKD patients.

Our study has several strengths. This is one of the largest studies of sarcopenia prevalence in PD patients. To our knowledge, this is the first study in ESKD patients to evaluate the individual concordance between older and recent internationally acknowledged definitions of sarcopenia, including SDOC, and the first to test the association of these criteria, as well as sarcopenia components, with functional limitation and dependency in CKD patients. We used standardized protocols to assess sarcopenia components to ensure the reproducibility of measurements.

There are several limitations to this study. This study is cross‐sectional in design and cannot assign the causality of sarcopenia to functional limitation. Our study enrolled consecutive patients from a single outpatient centre and excluded patients who could not stand or had other advanced diseases. This likely leads to an underestimation of the prevalence of sarcopenia and its association with disability. Nonetheless, our study cohort constitutes a sizable sample of fully or partially dependent PD patients, who represent the majority of patients on PD. The small number of patients with dependency constrained the statistical power of our multivariate analysis. While we could observe differences between diagnostic criteria, the limited sample size prevents robust statistical conclusions, particularly regarding the impact of severe sarcopenia. Finally, we used the bioimpedance technique for muscle mass estimation, which may produce different results than other methods, such as DEXA.

In conclusion, our study demonstrates a high sarcopenia prevalence in PD patients, accompanied by marked variations in the concordance and its association with functional limitation and dependency using different diagnostic criteria. Future longitudinal studies using clinically relevant outcomes are needed to identify optimal parameters and cutoff points to define sarcopenia in ESKD patients.

## Ethics Statement

The study was approved by the Institutional Review Board of the Faculty of Medicine, Ramathibodi Hospital, Mahidol University (COA. MURA # 2021/218 and #2023/821) and carried out according to the 1964 Declaration of Helsinki and its later amendments. All participants provided written informed consent. Details that might disclose individual patients have been omitted.

## Conflicts of Interest

The authors declare no conflicts of interest.

## Supporting information


**Table S1** Patient characteristics stratified by gait speed test completion status (completers, *n* = 301; non‐completers, *n* = 83).
**Table S2** Patient characteristics grouped by the presence or absence of sarcopenia according to the AWGS2019 definition.
**Table S3** Agreement between AWGS2019 and other diagnostic criteria for severity of sarcopenia (*n* = 384).
**Table S4** Risk factors associated with low muscle mass, low physical performance or low muscle strength by AWGS 2019 criteria (*n* = 384).
**Table S5** Comparison of ADL dependency (ADL score ≤11) according to sarcopenia components, classified by AWGS2019 cutoff criteria.
**Figure S1** Study flowchart.
**Figure S2** Patient‐level concordance among participants diagnosed with sarcopenia by at least one definition (*n* = 207). The Venn diagram illustrates the overlap and discrepancies across five sarcopenia definitions. Each number represents the patients classified as sarcopenic by the corresponding individual or combined criteria. Of note, only 15 patients fulfilled the sarcopenia diagnosis by all definitions, whereas 98 patients fulfilled the criteria for SDOC only.
**Figure S3** Odds ratios for functional impairment in each ADL domain associated with sarcopenia, defined by different criteria. Functional impairment was defined as a score below the full mark in each domain. Each bar compares patients with sarcopenia to those without (reference group). *p < 0.05.
**Figure S4** Prevalence of severe sarcopenia by different definitions. (a) All patients (*n* = 384); (b) age; age ≥ 65 years (*n* = 120), age < 65 years (*n* = 264) and (c) sex; men (*n* = 211) and women (*n* = 173).
**Figure S5** The proportion of patients with a functional limitation in the presence and absence of sarcopenia by different definitions and sarcopenia severity (*n* = 384). Functional limitation defined as Barthel Activities of Daily Living score ≤19.

## Data Availability

The datasets are available from the corresponding author upon reasonable request.
