# Supplementary material for: Recent Sarcopenia Definitions‐Variability in Prevalence and Disability Associations in Peritoneal Dialysis Patients
Source: J Cachexia Sarcopenia Muscle. 2025 Jul 29;16(4):e70018. doi: 10.1002/jcsm.70018 (PMC12304736; doi:10.1002/jcsm.70018)
Supplement: Supplementary file 1 — Table S1 Patient characteristics stratified by gait speed test completion status (completers, n = 301; non‐completers, n = 83). Table S2 Patient characteristics grouped by the presence or absence of sarcopenia according to the AWGS2019 definition. Table S3 Agreement between AWGS2019 and other diagnostic criteria for severity of sarcopenia (n = 384). Table S4 Risk factors associated with low muscle mass, low physical performance or low muscle strength by AWGS 2019 criteria (n = 384). Table S5 Comparison of ADL dependency (ADL score ≤11) according to sarcopenia components, classified by AWGS2019 cutoff criteria. Figure S1 Study flowchart. Figure S2 Patient‐level concordance among participants diagnosed with sarcopenia by at least one definition (n = 207). The Venn diagram illustrates the overlap and discrepancies across five sarcopenia definitions. Each number represents the patients classified as sarcopenic by the corresponding individual or combined criteria. Of note, only 15 patients fulfilled the sarcopenia diagnosis by all definitions, whereas 98 patients fulfilled the criteria for SDOC only. Figure S3 Odds ratios for functional impairment in each ADL domain associated with sarcopenia, defined by different criteria. Functional impairment was defined as a score below the full mark in each domain. Each bar compares patients with sarcopenia to those without (reference group). *p < 0.05. Figure S4 Prevalence of severe sarcopenia by different definitions. (a) All patients (n = 384); (b) age; age ≥ 65 years (n = 120), age < 65 years (n = 264) and (c) sex; men (n = 211) and women (n = 173). Figure S5 The proportion of patients with a functional limitation in the presence and absence of sarcopenia by different definitions and sarcopenia severity (n = 384). Functional limitation defined as Barthel Activities of Daily Living score ≤19. [file JCSM-16-e70018-s001.docx]

**ID: JCSM-D-24-00724)R2**

**Supplementary materials**

**Recent sarcopenia definitions- variability in prevalence and disability associations in peritoneal dialysis patients.**

Sasiwimon Meenetkum^1,2^, Sarinya Boongird^2^, Piyatida Chuengsaman^3^, Sirinapa Songsrakaew^2^, Sirarat Katesomboon^3^, Kanda Sriudom^3^, Prapimporn Chattranukulchai Shantavasinkul^4^, Wisit Chaveepojkamjorn^1^, Jiraluck Nontarak^1^, Chagriya Kitiyakara^2*^

^1^Department of Epidemiology, Faculty of Public Health, Mahidol University, Bangkok 10400, Thailand.

^2^Division of Nephrology, Department of Medicine, Faculty of Medicine Ramathibodi Hospital, Mahidol University, Bangkok 10400, Thailand.

^3^Banphaeo-Charoenkrung Peritoneal Dialysis Center, Banphaeo Dialysis Group, Banphaeo Hospital, Bangkok, Thailand.

^4^Division of Nutrition and Biochemical Medicine, Department of Medicine, Faculty of Medicine Ramathibodi Hospital, Mahidol University, Bangkok 10400, Thailand.

**Supplementary legends**

Table S1: Patient characteristics stratified by gait speed test completion status (completers, n=301; non-completers, n=83)

Table S2: Patient characteristics grouped by the presence or absence of sarcopenia according to the AWGS2019 definition.

Table S3: Agreement between AWGS2019 and other diagnostic criteria for severity of sarcopenia (n = 384).

Table S4: Risk factors associated with low muscle mass, low physical performance or low muscle strength by AWGS 2019 criteria (n=384).

Table S5: Comparison of ADL dependency (ADL score ≤11) according to sarcopenia components, classified by AWGS2019 cut-off criteria.

Figure S1: Study flowchart.

Figure S2: Patient-level concordance among participants diagnosed with sarcopenia by at least one definition (n=207).

Figure S3: Odds ratios for functional impairment in each ADL domain associated with sarcopenia, defined by different criteria.

Figure S4: Prevalence of severe sarcopenia by different definitions. a) All patients (n=384); b) Age; age ≥ 65 years (n=120), age < 65 years (n=264) and c) Sex; Men (n=211) and Women (n=173).

Figure S5: The proportion of patients with a functional limitation in the presence and absence of sarcopenia by different definitions and sarcopenia severity (n=384).

**Table S1: Patient characteristics stratified by gait speed test completion status (completers, n=301; non-completers, n=83)**

| **Characteristics** | **All Participants**  **(n=384)** | **Completers**  **(n=301)** | **Non-completers**  **(n=83)** | **P*-*value** |
| --- | --- | --- | --- | --- |
| **Demographic data**  Age (years) | 60.0 (52.0, 67.8) | 59.0 (50.5, 66.0) | 64.0 (54.0, 71.0) | **< 0.001*** |
| Weight (kg.) | 59.6 (52.2, 68.2) | 59.5 (51.9, 68.3) | 60.0 (52.8, 67.1) | 0.838 |
| BMI (kg/m^2^) | 23.0 (20.8, 26.0) | 23.1 (20.8, 26.0) | 22.7 (20.4, 25.4) | 0.457 |
| Married, *n* (%) | 227 (51.9) | 178 (59.1) | 49 (59.0) | 1.000 |
| Live alone, *n* (%) | 20 (5.2) | 15 (5.0) | 5 (6.0) | 0.519 |
| Education level, *n* (%)  - Primary school or lower | 208 (54.2) | 160 (53.2) | 48 (57.8) | 0.234 |
| - Secondary or vocational | 125 (32.6) | 104 (34.6) | 21 (25.3) |  |
| - Bachelor's degree or above | 51 (13.3) | 37 (12.3) | 14 (16.9) |  |
| Comorbidity, *n* (%)  - Hypertension | 345 (89.8) | 269 (89.4) | 76 (91.6) | 0.683 |
| - Diabetes mellitus | 271 (70.6) | 207 (68.8) | 64 (77.1) | 0.174 |
| - Cardiovascular disease | 180 (46.8) | 133 (44.2) | 47 (56.6) | **0.048*** |
| Peritoneal dialysis vintage (month) | 17.6 (8.5, 41.9) | 19.1 (8.5, 41.7) | 16.1 (8.1, 43.0) | 0.457 |
| **Laboratory parameters**  Hemoglobin (*n* = 378) | 10.4 (9.0, 11.6) | 10.3 (9.0, 11.5) | 10.5 (9.0, 12.0) | 0.213 |
| Serum C-Reactive Protein (mg/l) | 1.5 (0.5, 4.7) | 1.5 (0.5, 4.5) | 1.1 (0.4, 5.8) | 0.283 |
| Serum bicarbonate (mmol/L) | 24.3 (22.0, 26.6) | 23.9 (21.8, 26.6) | 24.3 (22.0, 26.5) | 0.477 |
| Serum blood urea nitrogen (mg/dL) | 44.3 (26.3, 60.0) | 45.8 (28.0, 62.3) | 41.6 (27.9, 58.9) | 0.236 |
| Serum creatinine (mg/dL) | 8.8 (4.1, 11.4) | 9.2 (4.4, 12.3) | 8.5 (3.9, 10.4) | 0.150 |
| Serum albumin (mg/dL) | 33.9 ± 7.7 | 34.1 ± 7.5 | 33.0 ± 5.5 | 0.426 |
| Serum Parathyroid hormone (pg/mL) | 165.6  (76.6, 317.2) | 177.1  (82.3, 336.7) | 127.7  (52.5, 211.7) | **0.002*** |
| Cholesterol (mg/dL) | 193.0  (158.0, 244.0) | 198.0  (159.0, 245.0) | 181.0  (152.0, 218.0) | 0.061 |
| Total Kt/V_urea_  (*n* = 363) | 2.1 ± 0.7 | 2.1 ± 0.6 | 2.2 ± 0.9 | 0.076 |
| Peritoneal_Kt/V_urea_ (*n* = 360) | 1.7 (1.5, 2.0) | 1.7 (1.4, 2.0) | 1.7 (1.5, 2.0) | 0.508 |
| nPNA (g/kg/day) (*n* = 355) | 1.1 (0.9, 1.3) | 1.1 (0.9, 1.3) | 1.1 (0.9, 1.3) | 0.566 |
| **Sarcopenia components**  ASM (kg) | 19.1 (15.7, 22.8) | 19.1 (15.5, 23.0) | 18.9 (16.6, 22.1) | 0.759 |
| ASMI (kg/m^2^) | 7.4 (6.5, 8.3) | 7.4 (6.4, 8.4) | 7.2 (6.6, 8.0) | 0.147 |
| ASM:BMI ratio | 0.8 (0.7, 1.0) | 0.9 (0.7, 1.0) | 0.8 (0.7, 1.0) | 0.179 |
| Handgrip strength (kg) (*n* = 379**) | 21.4 (16.5, 27.6) | 21.5 (16.9, 28.5) | 20.4 (14.2, 25.5) | **0.038*** |
| **Low muscle mass by ASM/ height^2^** (kg/m^2^), *n* (%)  AWGS2019  (Men < 7.0, Women < 5.7) | 87 (22.7) | 68 (22.9) | 19 (23.5) | 1.000 |
| EWGSOP2 (Men < 7.0, Women < 5.5) | 101 (26.7) | 79 (26.6) | 22 (27.1) | 1.000 |
| IWGS (Men < 7.23, Women < 5.67) | 93 (24.2) | 70 (23.6) | 23 (28.4) | 0.384 |
| **Low muscle mass by ASM: BMI**, *n* (%) |  |  |  |  |
| FNIH (Men < 0.789, Women < 0.512) | 42 (11.1) | 36 (12.1) | 6 (7.4) | 0.318 |
| **Low muscle strength,** *n* (%) ^a^  AWGS2019 (Men < 28, Women < 18) | 225 (58.6) | 163 (54.2) | 62 (74.7) | **< 0.001*** |
| EWGSOP2 (Men < 27, Women < 16) | 189 (49.2) | 137 (45.5) | 52 (62.7) | **0.006*** |
| FNIH (Men < 26, Women < 16) | 173 (45.1) | 124 (41.2) | 49 (59.0) | **0.004*** |
| SDOC (Men < 35.5, Women < 20^)^ | 311 (81.0) | 239 (79.4) | 72 (86.7) | 0.085 |
| **Low physical performance**, *n* (%) ^b^ |  |  |  |  |
| Gait speed < 0.8 m/s | 248 (64.6) | 166 (55.1) | 82 (98.8) | **< 0.001*** |
| Gait speed ≤ 0.8m/s | 198 (51.6) | 116 (38.5) | 82 (98.8) | **< 0.001*** |
| Gait speed < 1.0m/s | 198 (51.6) | 116 (38.5) | 82 (98.8) | **< 0.001*** |

* *P* < 0.05; Total Kt/VUrea represented the sum of weekly peritoneal and urine urea clearances; Peritoneal Kt/VUrea represented weekly peritoneal urea clearances; The body-mass index was calculated from weight in kilograms divided by height squared; BMI, body mass index; nPNA, normalized protein nitrogen appearance.a, Low muscle strength = unable to perform + handgrip strength (kg) by each criteria; b, Low physical performance= unable to complete + slow gait speed (ms) by each criteria

**Table S2: Patient characteristics grouped by the presence or absence of sarcopenia according to the AWGS2019 definition.**

| **Characteristics**  **AWGS2019** | **All cases**  **(n = 384)** | **Sarcopenia**  **(n = 86)** | **Non sarcopenia**  **(n = 298)** | ***P* value** |
| --- | --- | --- | --- | --- |
| **Demographic data**  Age > 65 years | 120 (31.3) | 40 (46.5) | 80 (26.8) | **0.001*** |
| Sex |  |  |  |  |
| - Men | 211 (54.9) | 48 (55.8) | 163 (54.7) | 0.902 |
| - Women | 173 (45.1) | 38 (44.2) | 135 (45.3) | 0.855 |
| BMI (kg/m^2^) | 23.1 (20.9, 26.0) | 20.4 (18.0, 22.5) | 23.7 (21.8, 27.0) | **< 0.001*** |
| Married, *n* (%) | 227 (59.1) | 52 (60.5) | 175 (58.7) | 0.804 |
| Live alone, *n* (%) | 20 (5.2) | 2 (2.3) | 18 (6.0) | 0.519 |
| Education level, *n* (%)  - Primary school or lower | 208 (54.2) | 54 (62.8) | 154 (51.7) | 0.162 |
| - Secondary or vocational | 125 (32.6) | 24 (27.9) | 101 (33.9) |  |
| - Bachelor's degree or above | 51 (13.3) | 8 (9.3) | 43 (14.4) |  |
| Income Patient(baht/month) |  |  |  |  |
| - > 10000 | 72 (18.8) | 10 (11.6) | 62 (20.8) | **0.002*** |
| - < 5000 | 253 (65.9) | 70 (81.4) | 183 (61.4) |  |
| - 5000 – 10000 | 59 (15.4) | 6 (7.0) | 53 (17.8) |  |
| Comorbidity, *n* (%)  - Hypertension | 345 (89.8) | 73 (84.9) | 272 (91.3) | 0.104 |
| - Diabetes mellitus | 271 (70.6) | 58 (67.4) | 213 (71.5) | 0.503 |
| - Cardiovascular disease | 180 (46.9) | 52 (60.5) | 218 (43.0) | **0.005*** |
| Peritoneal dialysis vintage (month) | 19.5 (9.3, 42.7) | 16.8 (9.1, 41.5) | 20.0 (9.6, 42.9) | 0.713 |
| **Laboratory parameters**  Hemoglobin (*n* = 378) | 10.4 (9.0, 11.6) | 11.1 (9.7, 12.1) | 10.3 (8.8, 11.4) | 0.118 |
| Serum C-Reactive Protein (mg/l) | 1.4 (0.5, 4.5) | 1.4 (0.6, 5.2) | 1.4 (0.5, 3.6) | 0.903 |
| Serum sodium (mmol/L) | 138.0  (136.0, 140.0) | 138.0  (136.0, 140.0) | 138.0  (136.0, 140.0) | 0.771 |
| Serum potassium (mmol/L) | 3.9 (3.4, 4.4) | 3.8 (3.3, 4.2) | 3.9 (3.4, 4.4) | 0.271 |
| Serum bicarbonate (mmol/L) | 24.0 (22.0, 26.6) | 23.7 (22.0, 26.1) | 24.3 (21.8, 26.6) | 0.377 |
| Serum blood urea nitrogen (mg/dL) | 44.8 (27.9, 60.5) | 41.6 (28.0, 54.6) | 46.8 (27.4, 62.8) | 0.295 |
| Serum creatinine (mg/dL) | 9.0 (4.2, 12.0) | 8.0 (5.4, 10.2) | 9.2 (3.9, 12.3) | 0.037* |
| Serum albumin (mg/dL) | 33.9 ± 7.7 | 34.3 ± 8.6 | 33.8 ± 7.4 | 0.674 |
| Serum Parathyroid hormone (pg/mL) | 166.8  (72.8, 318.5) | 172.0  (67.4, 285.2) | 164.6  (74.0, 320.0) | 0.903 |
| Cholesterol (mg/dL) | 193.0  (157.0, 242.5) | 183.0  (161.0, 232.5) | 196.0  (123.6, 244.0) | **0.049*** |
| Total Kt/V_urea_ (n = 363) | 2.1 ± 0.7 | 2.2 ± 0.7 | 2.1 ± 0.7 | 0.096 |
| Peritoneal_Kt/V_urea_ (n = 360) | 1.7 (1.5, 2.0) | 1.8 (1.5, 2.1) | 1.7 (1.5, 2.0) | 0.317 |
| nPNA (g/kg/day) (n = 355) | 1.1 (0.9, 1.3) | 1.1 (0.9, 1.3) | 1.1 (0.9, 1.3) | 0.590 |

* *P* < 0.05; Total Kt/VUrea represented the sum of weekly peritoneal and urine urea clearances; Peritoneal Kt/VUrea represented weekly peritoneal urea clearances; The body-mass index was calculated from weight in kilograms divided by height squared; BMI, body mass index; nPNA, normalized protein nitrogen appearance.

**Table S3: Agreement between AWGS2019 and other diagnostic criteria for severity of sarcopenia (n = 384).**

| **Diagnosis criteria** | **Level** | | | **Kappa test** | |
| --- | --- | --- | --- | --- | --- |
|  | **None sarcopenia** | **Sarcopenia** | **Severe sarcopenia** | **K^a^** | **p-value** |
| **AWGS2019 (ref)** | 298 (77.4%) | 33 (8.6%) | 53 (13.8%) |  |  |
| **EWGSOP2** | 311 (80.8%) | 25 (6.5%) | 48 (12.5%) | **0.83** | **<0.001*** |
| **FNIH** | 352 (91.4%) | 9 (2.3%) | 23 (6.0%) | **0.34** | **<0.001*** |

a The Kappa value greater than (0.75) represent good; (0.40-0.75) moderate agreement, (<0.40) suggest poor agreement beyond chance; AWGS2019, the Asian Working Group for Sarcopenia 2019; EWGSOP2, the European Working Group on Sarcopenia in Older People 2; FNIH, the Foundation for the National Institutes of Health.

**Table S4: Risk factors associated with low muscle mass, low physical performance or low muscle strength by AWGS 2019 criteria (n=384).**

|  | **Low muscle mass** | | | | **Low physical performance** | | | | **Low muscle strength** | | | |
| --- | --- | --- | --- | --- | --- | --- | --- | --- | --- | --- | --- | --- |
| **Variables** | **Univariate OR**  **(95%CI)** | ***P* value** | **Multivariate OR**  **(95%CI)** | ***P* value** | **Univariate OR**  **(95%CI)** | ***P* value** | **Multivariate OR**  **(95%CI)** | ***P* value** | **Univariate OR**  **(95%CI)** | ***P* value** | **Multivariate OR**  **(95%CI)** | ***P* value** |
| Age (years) | **1.03**  **(1.01 – 1.05)** | **0.010*** | **1.04**  **(1.01 – 1.06)** | **0.003*** | **1.04**  **(1.02 – 1.05)** | **<0.001*** | **1.03**  **(1.01 – 1.05)** | **< 0.001*** | **1.05**  **(1.03 – 1.01)** | **< 0.001*** | **1.05**  **(1.03 – 1.06)** | **< 0.001*** |
| Sex (- Men (Ref) vs Women | 0.92  (0.57 – 1.49) | 0.739 |  |  | 1.22  (0.80 – 1.86) | 0.360 |  |  | 0.90  (0.60 – 1.36) | 0.622 |  |  |
| Body mass index (kg/m^2^) | **0.67**  **(0.61 – 0.75)** | **< 0.001*** | **0.64**  **(0.57 – 0.73)** | **< 0.001*** | 1.00  (0.95 – 1.05) | 0.886 |  |  | **0.95**  **(0.91 – 1.00)** | **0.049*** | **0.94**  **(0.90 – 1.00)** | **0.016*** |
| Hypertension  (No (Ref) vs Yes) | **0.34**  **(0.17 – 0.67)** | **0.002*** | **0.24**  **(0.10 – 0.61)** | **0.003*** | 1.47  (0.75 – 2.87) | 0.262 |  |  | 0.87  (0.44 – 1.72) | 0.694 |  |  |
| Diabetes  (No (Ref) vsYes) | 0.68  (0.41 – 1.14) | 0.145 |  |  | 1.18  (0.75 – 1.85) | 0.486 |  |  | **1.69**  **(1.08 – 2.63)** | **0.021*** | **1.70**  **(1.03 – 2.68)** | **0.038*** |
| Cardiovascular disease (No (Ref) Vs Yes | **1.66**  **(1.03 – 2.70)** | **0.038*** | 1.77  (0.97– 3.24) | 0.061 | 1.50  (0.98 – 2.29) | 0.062 | 1.44  (0.93 – 2.22) | 0.101 | **1.80**  **(1.19 – 2.73)** | **0.005*** | **1.70**  **(1.08 – 2.59)** | **0.020*** |
| Peritoneal dialysis vintage (months) | 1.00  (0.99 – 1.01) | 0.755 |  |  | 1.00  (0.99 – 1.01) | 0.695 |  |  | 1.00  (1.00 – 1.01) | 0.423 |  |  |
| Serum creatinine (mg/dL) | 0.96  (0.91 – 1.00) | 0.075 | 0.96  (0.91 – 1.03) | 0.273 | 0.96  (0.92 – 1.00) | 0.059 | 0.98  (0.94 – 1.02) | 0.321 | 0.97  (0.93 – 1.01) | 0.176 |  |  |
| Serum albumin (mg/dL) | 1.02  (0.98 – 1.05) | 0.315 |  |  | 1.00  (0.97 – 1.03) | 0.894 |  |  | **0.97**  **(0.95 – 1.00)** | **0.041*** | 0.98  (0.95 – 1.01) | 0.158 |
| Total Kt/Vurea | 1.34  (0.95 – 1.88) | 0.095 | 1.33  (0.82 – 2.15) | 0.252 | 1.14  (0.82 – 1.59) | 0.437 |  |  | 0.91  (0.67 – 1.24) | 0.571 |  |  |
| nPNA (g/kg/day) | 0.97  (0.84 - 1.12) | 0.697 |  |  | 1.01  (0.95 – 1.08) | 0.650 |  |  | 1.04  (0.91 – 1.18) | 0.590 |  |  |

Risk factors associated with the presence of low muscle mass, low physical performance or low muscle strength by AWGS 2019 cut-off criteria versus those with normal parameter.

Total Kt/V_Urea_ represented the sum of weekly peritoneal and urine urea clearances; nPNA, normalized protein nitrogen appearance.

**Table S5: Comparison of ADL dependency (ADL score ≤11) according to sarcopenia components, classified by AWGS2019 cut-off criteria.**

| **Sarcopenia component**  **By AWGS2019** | **ADL dependency status**  ***n* (%)** | | **P-value** |
| --- | --- | --- | --- |
|  | **Independent** | **Low to moderately dependent** |  |
| **Muscle strength**  Low muscle strength | 212 (94.2) | 13 (5.8) | **0.002*** |
| Normal muscle strength | 159 (100.0) | 0 (0.0) |  |
| **Muscle mass**  Low muscle mass | 87 (100.0) | 0 (0.0) | 0.126 |
| Normal muscle mass | 282 (96.9) | 9 (3.1) |  |
| **Physical performance**  Low physical performance | 237 (95.6) | 11 (4.4) | 0.150 |
| Normal physical performance | 134 (98.5) | 2 (1.5) |  |

* *P* < 0.05; Chi-square test; ADL, Activities of Daily Living; AWGS2019, the Asian Working Group for Sarcopenia 2019.

**Supplementary Figures**

**Figure S1: Study flowchart.**

Patients undergoing peritoneal dialysis were assessed for eligibility (n=414)

Excluded (n=30)

- declined to participate (n=15)

- withdrew from the study (n=2)

- unable to undergo BIA: limb amputation, unable to stand, or had pacemaker (n=10)

- inability to draw blood (n=1)

- life expectancy less than 6 months (n=2)

Patients undergoing peritoneal dialysis were included into the study (n=384)

**Figure S2:** **Patient-level concordance among participants diagnosed with sarcopenia by at least one definition (n=207).** The Venn diagram illustrates the overlap and discrepancies across five sarcopenia definitions. Each number represents the patients classified as sarcopenic by the corresponding individual or combined criteria. Of note, only 15 patients fulfilled the sarcopenia diagnosis by all definitions, whereas 98 patients fulfilled the criteria for SDOC only.


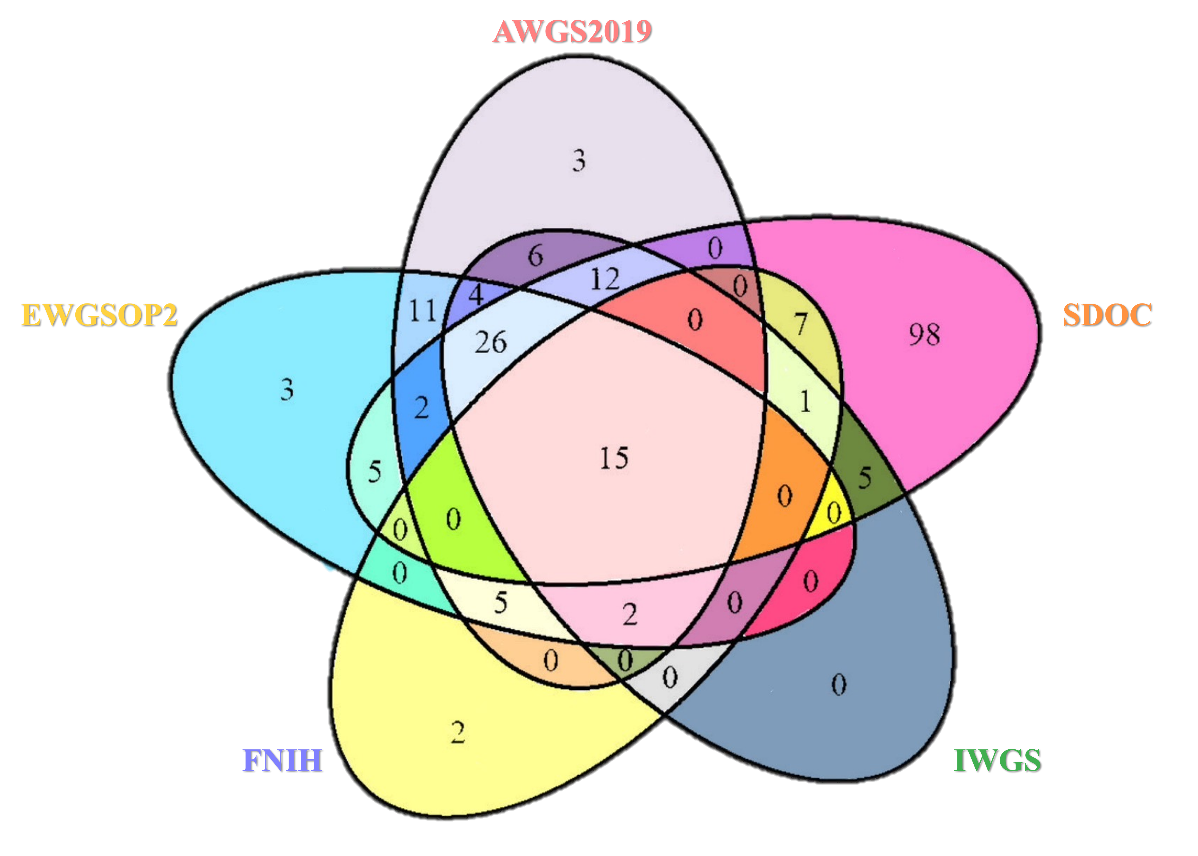


AWGS2019, the Asian Working Group for Sarcopenia 2019; EWGSOP2, the European Working Group on Sarcopenia in Older People 2; FNIH, the Foundation for the National Institutes of Health; IWGS, the International Working Group for Sarcopenia; SDOC, the Sarcopenia Definitions and Outcomes Consortium

**Figure S3****:** **Odds ratios for functional impairment in each ADL domain associated with sarcopenia, defined by different criteria.** Functional impairment was defined as a score below the full mark in each domain. Each bar compares patients with sarcopenia to those without (reference group). *P <0.05.


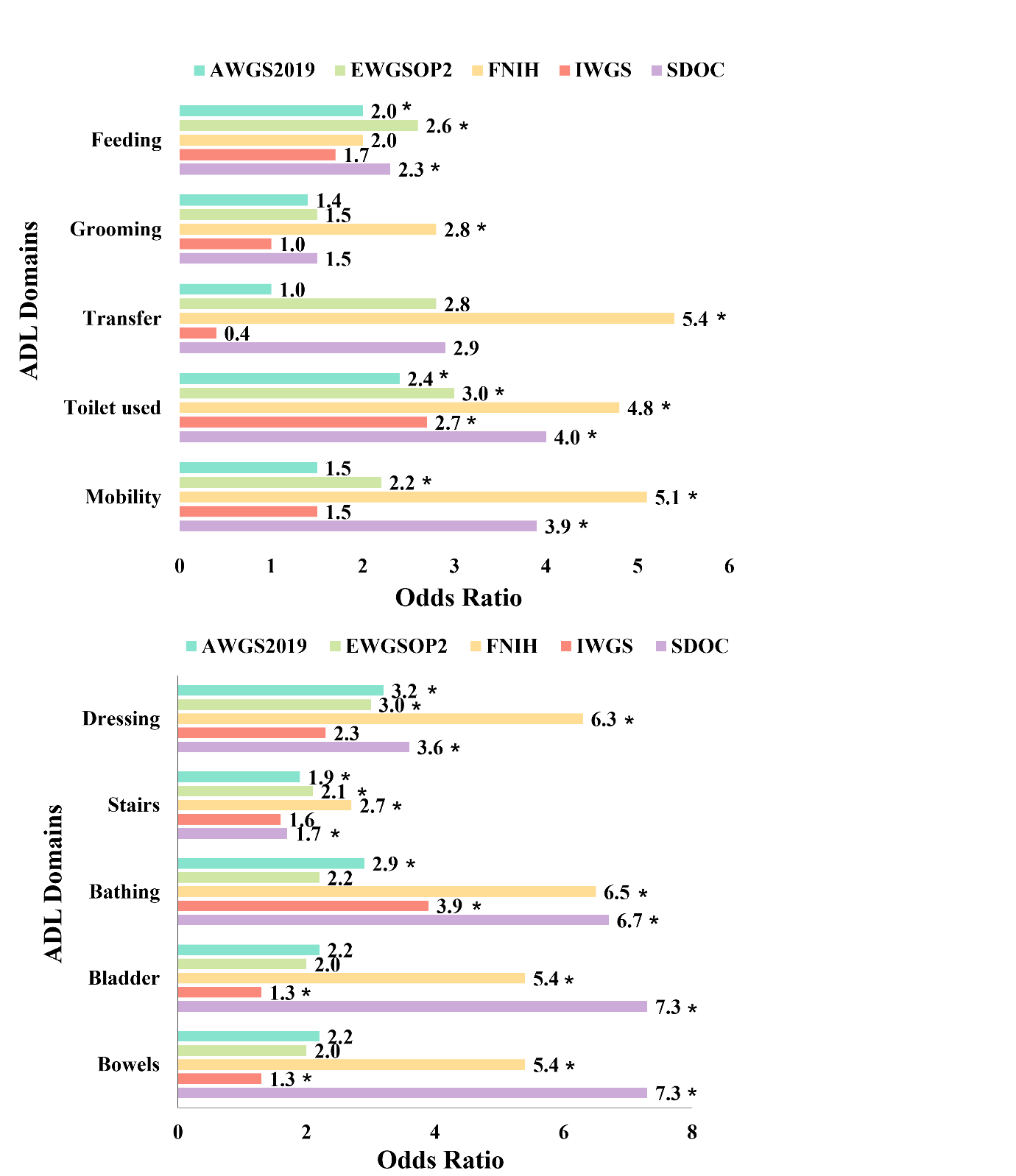


AWGS2019, the Asian Working Group for Sarcopenia 2019; EWGSOP2, the European Working Group on Sarcopenia in Older People 2; FNIH, the Foundation for the National Institutes of Health; IWGS, the International Working Group for Sarcopenia; SDOC, the Sarcopenia Definitions and Outcomes Consortium

**Figure S4:** **Prevalence of severe sarcopenia by different definitions. a) All patients (n = 384); b) Age; age ≥≥ 65 years (n = 120), age < 65 years (n = 264) and c) Sex; Men (n = 211) and Women (n = 173).**

**
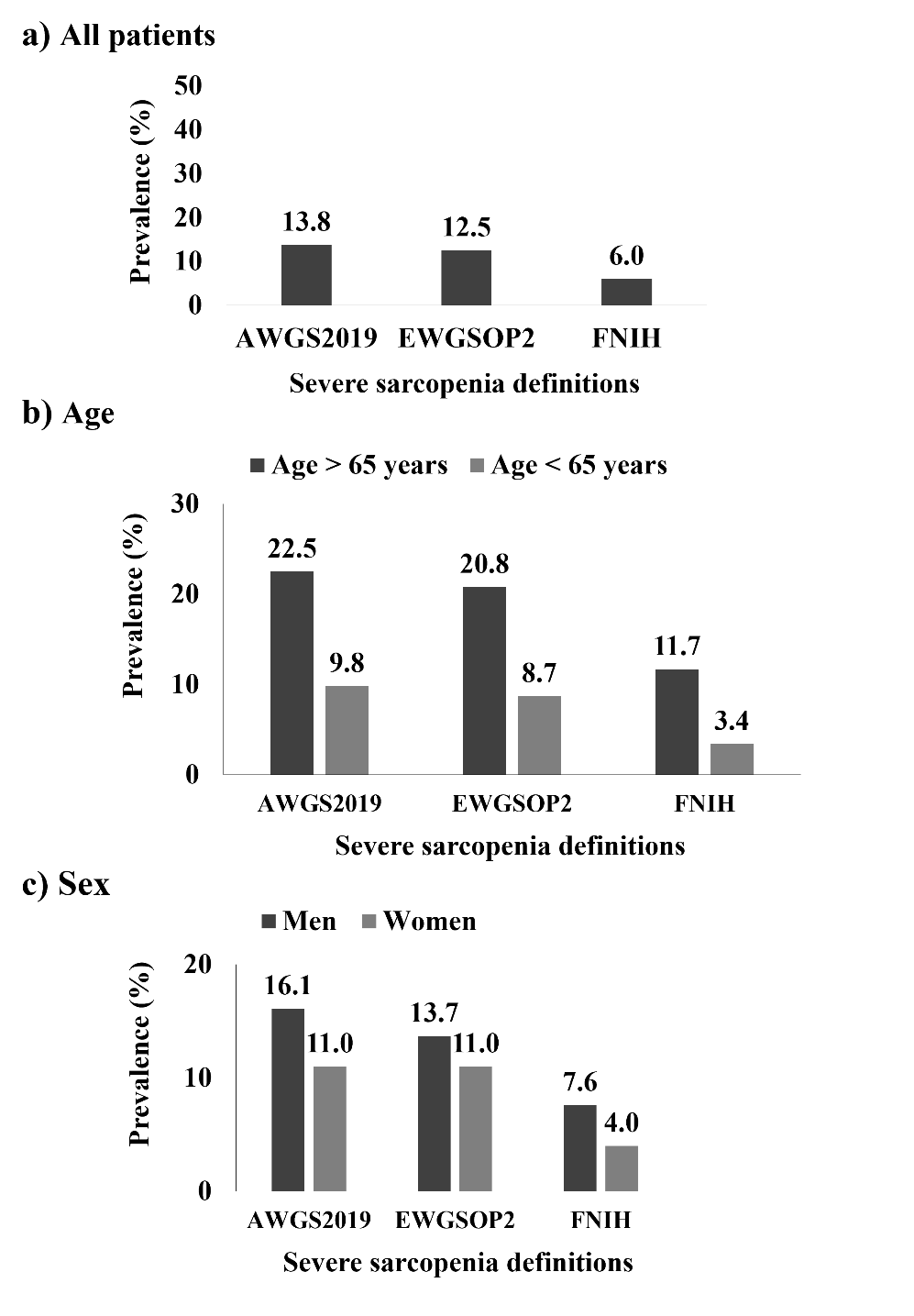
**

AWGS2019, the Asian Working Group for Sarcopenia 2019; EWGSOP2, the European Working Group on Sarcopenia in Older People 2; FNIH, the Foundation for the National Institutes of Health; IWGS, the International Working Group for Sarcopenia; SDOC, the Sarcopenia Definitions and Outcomes Consortium

**Figure S5**: **The proportion of patients with a functional limitation in the presence and absence of sarcopenia by different definitions and sarcopenia severity (n=384).** Functional limitation defined as Barthel Activities of Daily Living) score ≤19.


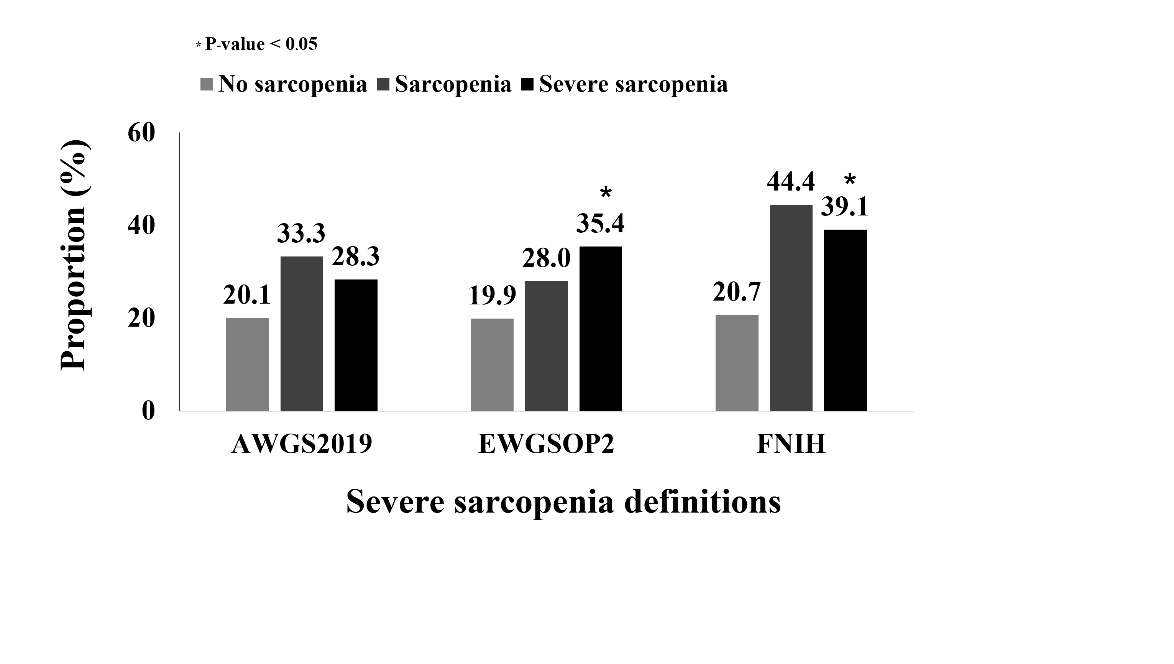


AWGS2019, the Asian Working Group for Sarcopenia 2019; EWGSOP2, the European Working Group on Sarcopenia in Older People 2; FNIH, the Foundation for the National Institutes of Health.
